# A Study on the Adoption of Blockchain for IoT Devices in Supply Chain

**DOI:** 10.1155/2022/9228982

**Published:** 2022-07-19

**Authors:** Muhammad Anas Baig, Danish Ali Sunny, Abdullah Alqahtani, Shtwai Alsubai, Adel Binbusayyis, Muhammad Muzammal

**Affiliations:** ^1^Department of Computer Science, Bahria University, Islamabad 44000, Pakistan; ^2^Department of Applied Mathematics Statistics, Institute of Space Technology, Islamabad 44000, Pakistan; ^3^College of Computer Engineering and Sciences, Prince Sattam bin Abdulaziz University, Al-Kharj 11942, Saudi Arabia; ^4^Department of Software Engineering, Bahria University, H-11 Campus, Islamabad 44000, Pakistan

## Abstract

The integration of blockchain and IoT enables promising solutions in decentralized environments in contrast with centralized systems. The blockchain brings forth features such as fault tolerance, security, and transparency of the data in IoT devices. As there is requirement of consensus among the network nodes to agree on a single-state-of-ledger, nonetheless, the extensive computational requirement for the consensus protocol becomes a limitation in resource-constrained IoT devices with limited battery, computation, and communication capabilities. This study proposes an empirical approach on the adoption of blockchain in a supply chain environment. Furthermore, a modified version of the Raft consensus protocol is proposed for use in supply-chain environment on the permissioned blockchain Hyperledger. In Raft consensus protocol, each transaction is directed to the leader node that transmits it to the follower nodes, making the leader node the bottleneck thus inhibiting the scalability and throughput of the system. This also results in high latency for the network. The modified RAFT consensus protocol (mRAFT) is based on the idea of utilizing the idle follower nodes in disseminating the vote requests and log replication messages. A detailed empirical evaluation of the solution built on Hyperledger Caliper is performed to demonstrate the applicability of the system. The improved workload division on the peers boosts the throughput and latency of the system in ordering service that enhances the overall efficiency of the system.

## 1. Introduction

The Internet of Things (IoT) is an emanating technology with a massive impact on day-to-day activities of either an individual for his routine tasks or organizations for managing and proposing novel business models. Due to the emergence of self-intelligent computing machines, IoT is shifting from automatic to autonomous systems where intelligent smart devices could learn from their environment and make decisions accordingly, revolutionizing the ubiquitous digital environment towards a new era. IoT has become part of every aspect of the modern world, from intelligent healthcare systems to intelligent transportation systems, traffic control systems, supply chain management, smart grid, and many others in which several heterogeneous devices communicate with each other to provide an intelligent digital environment. IoT devices generate a massive amount of data, and all intelligent decisions are solely based on the sensed data, which must be accurate and pure from any mutations.

As a means of communication, IoT devices typically use centralized client-server architecture in which all communication is done through the backbone network of cloud that is not much suitable to scale, manage, and control a large number of IoT devices. Moreover, there is a considerable overhead in managing diverse nature of heterogeneous devices that need to communicate efficiently in a delay critical manner. The centralized architecture of cloud computing in IoT is severely prone to a single point of failure, which is vulnerable to system collapse and inhibits an environment in which numerous IoT devices could communicate seamlessly; besides that, cloudis expensive to manage and maintain. Traditional IoT network never fits in the delay-critical environment for resource-constrained devices based on storage and power. Even if devices are near each other, they still need to communicate through a long distant cloud server. Considering the downside of the traditional IoT network centralized architecture, there is a need for a system that provides a decentralized, fault-tolerant, secure, and widely scalable mechanism to manage IoT network infrastructure in which devices induce heterogeneity among systems and trust is managed between them.

Blockchain technology surprisingly solves most of the significant issues of centralized IoT network architecture. It provides a secure, trustless, transparent, traceable, scalable, and highly fault-tolerant environment for IoT devices communication. Blockchain technology is currently one of the most discussed technologies that eliminated the need of the third party for validating the transactions using the consensus mechanism of existing nodes over the peer-to-peer network [1]. A blockchain is analogous to a database that signifies the data definition in parallel to the data update mechanism; blockchain allows to add new data and ensures that the state of the data on all nodes over the peer-to-peer network is alike. Blockchain also maintains the historical state of the data and ensures that such rules are enforced in which linked blockchain structure is resistant to any modification [2]. Via reverse engineering blockchain technology, it could be seen that blockchain is just the combination of old technologies in a different manner, in which core technologies include cryptography, distributed ledger technology, peer-to-peer network, and few others [3].

**Figure 1:**
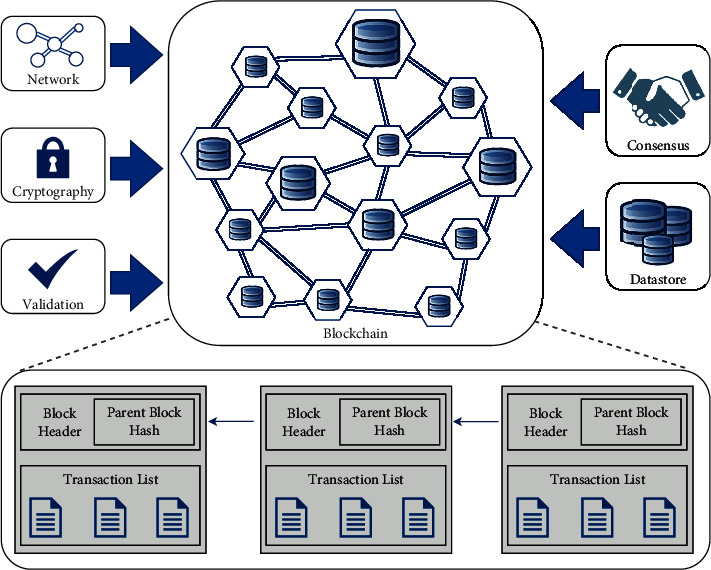
Basic components of a blockchain architecture.

A blockchain is a singleton machine with a shared data state that has a very precise principal architecture [4]. Blockchain architecture comprises of components like “cryptography” to ensure security and integrity of transactions, “datastore” to store the data in a decentralized network, “validation” to determine the transaction conforms to protocol rules, “peer-to-peer network” of nodes running blockchain protocol and “consensus” mechanism to achieve the significant agreement on a single state of the ledger, as described in Figure 1. Blockchain is the consecutive sequence of blocks, consisting of block-header and block-body. Block body contains numerous transaction records in a public ledger. Each block header contains the block number and previous block hash; however, the first block has no previous or parent block, hence called as genesis block [5].

Bitcoin [6] as the first use case of blockchain technology is a digital currency synonymous with money, the intent behind Bitcoin was to make cross border payments secure and transparent, which facilitates two parties located globally to transact without any expensive intermediary. This whole payment system is solely based on secure cryptographic evidence. However, Bitcoin blockchain, also referred to as Blockchain-1.0, has a very limited scripting language obstructing the development of applications on top of the Bitcoin blockchain. Bitcoin blockchain limiting application development over it sparked the emergence of turing-complete Ethereum blockchain. Ethereum blockchain, also referred to as the world computer and Blockchain-2.0, supports running any sort of computational code via smart contracts in a completely decentralized fashion, maintaining a singleton state of data across the ledger [7].

Beyond Blockchain-2.0, private blockchains came into existence that authenticates only the known entities in the blockchain; private blockchains offer numerous data processing capabilities as the study [8] is a detailed discussion on the data processing view of blockchain systems. Enterprise level blockchains offer several benefits as one of the popular permissioned blockchains; Hyperledger is an open-source collaborative effort that enables cross-industry blockchain applications for business organizations. Among numerous flavours of Hyperledger, Fabric is one of the popular permissioned blockchains that provides a foundation for private enterprise-owned blockchain-based solutions, products, and software applications [9]. Corda is another blockchain platform that supports the creation of distributed applications on top of it for usage across the financial sector, insurance, and trade finance [10]. Based on public blockchain Ethereum, Quorum is a permissioned blockchain that is implemented to support the enterprise needs of Ethereum blockchain [11]. Also, there are many scenarios where blockchain technology is implementable. With time, research is expanding on enterprise-grade permissioned blockchain technology, opening the door to surprising facts of innovating the traditional organizational structures with blockchain technology.

Considering the tremendous benefits of blockchain and its hybrid with IoT could solve many problems of IoT like trust, security, and maintenance cost problems. Blockchain provides a peer-to-peer network instead of relying on a single cloud or data center. Blockchain is not vulnerable to a single point of failure; furthermore, privacy and security are ensured via cryptographic hashing algorithms, solving reliability issues. The blockchain technology used in IoT will let the network execute autonomous transactions, storing business logic in smart contracts. Similarly, the public ledger of the blockchain will enable IoT devices to ensure transparency among their operations and let the stakeholders audit the information of IoT devices [12]. Decentralization in the blockchain is achieved through consensus between nodes without the involvement of a third party which is the most significant feature of blockchain for IoT. The hybrid of blockchain and the IoT is questioned by its computationally intensive cryptographic hashing algorithms in consensus methods and delay in transactions, or their confirmations that are inevitable; hence lightweight consensus methods with low latency and high computational efficiency are required for blockchain-enabled IoT networks [13]. This study analyses the different consensus methods in blockchain, their applicability in the scenario of resource-constrained IoT devices provides the improved consensus mechanism mRAFT for IoT devices-based blockchain networks. IoT devices are power and storage constrained, limiting the use of blockchain in IoT networks as blockchain involves computationally intensive hashing algorithms in consensus methods. The consensus algorithms used in blockchain are not built considering the capability constraints of IoT devices. To address this issue, there is a need to develop IoT resource-friendly consensus algorithm that utilizes the idle resources in blockchain peer nodes to assist the IoT devices in reaching consensus efficiently.

Internet of Things (IoT) devices are becoming part of every day; innovative systems like smart homes, intelligent transportation systems, and smart supply chains are emerging rapidly. In the centralized architecture of IoT devices network, there are issues like security, maintenance cost, less fault tolerance, and many others. On the other hand, Blockchain technology is one of the most researched technologies in computer science that eliminated the need for the centralized third party for performing transactions globally. Hybrid of the IoT and blockchain technology is worthful because it solves centralized IoT devices network issues like transparency, security, fault tolerance, and auditability needs of the IoT devices. However, the blockchain needs the consensus between the nodes in the network to agree on the single state of the blockchain data. Integrating IoT devices over a blockchain network is massively beneficial, but the resource-intensive computation required by the blockchain consensus mechanism is overhead for resource-constrained IoT devices. The power constraint nature of IoT devices makes it a wrong choice for running computationally intensive consensus algorithms of blockchain. Due to this fact, there is a significant challenge to analyze, determine, and develop such consensus algorithms that are resource-friendly for IoT resource-constrained devices and provide the best possible consensus among IoT devices with insignificant trade-offs to bear. Moreover, there is huge scale usage of IoT devices in supply chain management. There is a significant need to analyze the hybrid of blockchain and IoT in the supply chain sector to optimize the performance of IoT devices and blockchain frameworks as per the supply chain management needs. This research aims to study different consensus methods in blockchain, analyze their applicability for IoT resource-constrained devices and ultimately propose the improved consensus algorithm for IoT devices network. Furthermore, we finally explore the hybrid architecture of permissioned blockchain and IoT devices network in the supply chain sector.

Blockchain belongs to various categories based on its controlling authority and read/write access. Public vs. private refers to the write access rights of the blockchain. Open vs. closed means which entities could read data from the blockchain. While permissioned vs. permissionless blockchain tells whether only entities with special access permissions could interact with the blockchain or everyone can connect and make transactions over the blockchain. The scope of this research is focused on permissioned blockchain Hyperledger Fabric and specifically its Raft-based consensus algorithm for its ordering service. The research analyzes the applicability of blockchain technology in IoT resource-constrained device networks and thoroughly analyzes the different blockchain consensus methods. Finally, it proposes an improved consensus mechanism for IoT resource-constrained devices network and subsequently evaluates its application in a blockchain-based supply chain case study. As one of the primary usages of IoT devices is in the supply chain sector, exploring the efficiency of the proposed improved raft algorithm in reference to the supply chain case study let this research thoroughly analyze all the shortcomings of the proposed algorithm concerning the real-life application. The research constraints of this study include the assumption that the system could share signatures with the devices over the network in a secure manner. The system admin is a trusted person who initializes the processes at first, and all actions carried out by the admin are legitimate.

The literature on the blockchain is reviewed in the first phase, which identifies the transition of the public blockchain toward the permissioned blockchain. In the second phase, literature related to blockchain-based IoT is studied, signifying why and why not public blockchains are suitable for IoT. Public blockchains are best suitable for environments that involve public use of IoT devices openly. However, industrial IoT does not need everything to be open and accessible to everyone. IoT for enterprise requirements needs identifiable and authorized participants, high transaction throughput performance requirements, and most importantly, the confidentiality of the private transactions and data. The permissioned blockchain of enterprise-grade Hyperledger Fabric architecture is reviewed, including thoroughly studying its modular architecture, transaction flow, certificate management, and ordering service. In the third phase, the core of our research, “consensus methods in blockchain-based IoT,” is reviewed. Whitelisting of the nodes needed for consensus in a distributed network is done in some cases to make consensus lightweight, which raises security concerns for the decentralized system. IoT could be managed with centralized architecture; however, significant issues could be solved by introducing decentralization in IoT network architecture. The literature review order of sequence helped understand the blockchain architecture and different blockchain solutions available. Secondly, making the perception about the hybrid of IoT and blockchain technology and thirdly developing an in-depth understanding of the commonalities and variances and pros and cons among numerous consensus methods in blockchain-driven IoT networks.

Raft is a strong leader-based consensus protocol in which all the operations are done via the leader node. In our mentioned IoT-based supply chain scenario, follower nodes, upon receiving the messages, disseminate the messages to a further subset of nodes, due to which finally the message is propagated through the whole network. It is important to note here that the term number attached to each message lets the follower nodes determine which version of the message they are receiving, so if the message received is from the older term, then it is automatically rejected by the follower nodes. In this way, the improved version mRAFT of the raft algorithm in Hyperledger Fabric makes use of the idle follower nodes and reduces the workload from the leader node. In return, leader nodes utilize the saved time to validate and propagate the remaining transactions. The balanced workload on every peer improves the overall performance and efficiency of the Raft consensus in Hyperledger Fabric.

This paper has the following key contributions.

- The literature related to the applicability of blockchain technology in solving traditional IoT devices network limitations is thoroughly studied and analyzed.
- The characteristics of different blockchain-based consensus algorithms in IoT are analyzed which helped in developing an in-depth understanding of the commonalities and variances and pros and cons among numerous consensus methods in blockchain-driven IoT networks.
- An improved consensus algorithm in terms of latency and throughput mRAFT which uses idle follower nodes for message dissemination is developed for IoT-enabled blockchain and analyzed mRAFT algorithm in reference to blockchain-based supply chain case study.
- The related issues in performance bottleneck and challenges in IoT-enabled blockchain to which succeeding research could be directed are identified.

The structure of the paper is as follows.  Section 2 outlines the recent related work.  Section 3 introduces the proposed technique, while Section 4 gives the evaluation of the proposed methodology. Finally, Section 5 concludes the findings of the work done and identifies the future directions.

## 2. A Review of the State of the Art

Blockchain is a discovery of the recent past, and several investigations were conducted to evaluate the usage of public blockchains in traditional applications. Public blockchains research is transitioning towards permissioned or private blockchains as it is a more exciting research area for big enterprise organizations. Permissioned blockchains research work is reviewed to analyze how it could fit in the IoT devices network architecture scenario. Furthermore, blockchains and their consensus methods application in the network of resource-constrained IoT devices based on storage and power consumption are also analyzed. The main aim of this research is to explore different available blockchain solutions and then optimize the blockchain algorithms for use in IoT device networks. In reviewing the state of the art, firstly the literature related to the public blockchain is discussed which also signifies the emerging need for permissioned blockchains for organizational use cases. After that, the literature related to the use hybrid of blockchain and IoT is discussed which explains the use of public blockchains in IoT and its transition to permissioned blockchain for use in industrial-IoT systems. There is a need for identifiable and authorized participants in industrial IoT therefore the permissioned blockchain is the most useful. For the sake of permissioned blockchain, the enterprise-grade Hyperledger Fabric is thoroughly reviewed, discussing its modular architecture, ordering service, and transaction flow. The purpose of discussing the Hyperledger Fabric is to dive deep into its architectural modules and consensus method so that the challenging issues could be resolved in the proposed mRAFT consensus algorithm. Lastly, the literature related to consensus methods in blockchain-driven IoT is discussed. The review of the different blockchain-based consensus algorithms for IoT provides strong theoretical groundings on the optimization of the proposed mRAFT consensus algorithm. There is also a concept reviewed in literature in which IoT devices resource-constrained nature smart homes are loaded with high-performance computing resources to provide additional resources for the computational tasks of IoT resource-constrained devices such that providing them support in undergoing lightweight consensus among IoT devices. The studies discussing the core behind consensus mechanisms PoW, PoS, and DAG-based Tangle by IOTA blockchain revealed the challenges and applicability of DAG-based consensus mechanisms in blockchain-driven IoT. Moreover, the literature review revealed strategies that introduce rewards in consensus algorithms that ultimately proposed the evolution of credit-based consensus mechanisms that work by increasing the computational workload for malicious nodes while decreasing the workload for honest nodes.

### 2.1. Permissioned Blockchain

Vukolić [14] identified that current permissioned blockchains have architectural limitations like all nodes must execute the smart contracts, contracts run sequentially, non-determinism exists in smart contract execution, and rigid consensus mechanisms that are not suitable for every use case. The paper discusses that the nondeterminism in smart contracts in the public blockchain is handled by “gas” that limits the execution capacity as per the gas amount; the more gas the smart contract has, the more is its execution capacity. The gas feature is applicable due to the inherent Ether currency in the Ethereum blockchain, where gas could be bought via paying in Ether currency. However, no such payment mechanism is defined in permissioned blockchains, especially Hyperledger, which does not have inherent cryptocurrency. In the context of the addressed issues, the paper proposed the redesign of the architecture of Hyperledger Fabric, introducing the modification to execute-order-validate architecture and providing flexible endorsement policies. However, this paper did not provide the mechanism to translate and generate chaincodes from policies specified in the policy language.

Risius and Spohrer [15] recognized that most of the time, the technical applicability and practical benefits of blockchain are neglected, concerning respective issues critics consider blockchain technology as overhyped. The paper elaborated the techniques for structuring blockchain advancements, proposed the research framework for blockchain systems, and finally provided insights on avenues for blockchain advance research in multiple disciplines to help blockchain research sustain instead of getting overhyped. However, this research lacks the consideration of the technical aspects in providing the multidisciplinary research approach in the blockchain.

Saraf and Sabadra [16] discussed technical aspects of Ethereum blockchain-like currency, transaction processing, gas price, and consensus algorithm. The research then contrasted the Hyperledger flavors - Burrow, Fabric, Indy, Iroha, and Sawtooth based on maturity status, consensus algorithm, REST API, interoperability scale, and programming languages support and finally, contrasting Ethereum, Hyperledger and Corda blockchain based on platform type, governance, mode of operation, consensus, currency, contract language, and data storage. The research inferred that Hyperledger is best where security and privacy are the priority. At the same time, the Ethereum blockchain is best for processing transactions with decentralized miners, inhibiting third-party involvement. However, the Corda blockchain can be used in an environment where financial agreements are essential to business processes. However, the research lacks the insights on most significant performance metrics such as block creation time, block size, cost and power consumption.

Lasisi and Hsu [17] described how permissionless blockchain consensus evolved in permissioned blockchain. PBFT in Bitcoin includes two validation processes, the consensus from the mining node and then from all the miner nodes for verifying and publishing the block to the blockchain. With validation process reduction to one Ethereum blockchain changes PBFT to Smart Contract, a self-executing business logic when certain conditions are met. Hyperledger evolved the Smart Contract to Chaincode in which all the consensus is done via ordering service. The research investigates and concludes that the consensus mechanism is the rationale behind discussed evolution. However, the major limitation of this research is that it did not address the significant modular architectural differences of Hyperledger from the previous blockchains.

Nadir [18] contributed towards contrasting the permissioned blockchain leading houses Fabric, Corda, and Quorum blockchain based on comparison filters of modularity, language support, privacy, transactions per second, currency support, and adaptability. The comparison filters are scaled between 1 and 3 and computing the final score, representing the overall adaptability of the framework. The research winded up by computing the results that deduced Fabric with high adaptability score due to its modular structure and pluggable ordering service. Similarly, the evaluation of Corda blockchain resulted in a second adaptability score that leads ahead of Quorum blockchain due to its latest security feature of the hardware security module. Then, it surmises Quorum blockchain with the least general adaptability score due to its rigid and less adaptable architecture. This paper ignored the crucial performance comparison metrics like block creation time, block size, and energy consumption.

Ban et al. [19] empirically examined the frameworks under the Hyperledger family consisting of Fabric, Sawtooth, Iroha, Burrow, and Indy by contrasting them based on three layers; data model layer (transactions/smart contracts, blocks), execution layer (nodes/blockchain), and consensus layer (consensus, DLT). The experimental design has qualitative comparison metrics as interfaces, network deployment, transaction hashes, transaction privacy, smart contracts, consensus, zero-knowledge proof, access control, private DB within a chain, block size, release frequency, and license. The quantitative comparison is based on benchmarking and performance evaluation to measure latency and throughput on different node configurations. The research paper concluded by presenting the score-based comparison of different characteristics of Hyperledger flavours that will help the programmer choose the best among them as per their use case scenario. However, this research did not address the cost-effectiveness of maintaining each scheme and the power consumption of managing the peers of the network.

Thota et al. [20] discussed one of the significant concerns in blockchain technology that is preserving and securing the private keys for the end-user in a software wallet to give the end-user control of its identity. In the blockchain, assets are secured by using the Public Key Infrastructure (PKI). Only the entity having access to the secret private keys could undergo the transactions on his asset. In the proposed scheme, two participants, regulatory authorities and organizations, require KYC information for end-users. Blockchain identity is issued for the end-user in which his transaction is recorded as “recordConsent” on the backend of which Hyperledger Fabric smart contract executes. The research proposed the implementation of a software wallet on top of Hyperledger Fabric to enhance security in enterprise applications and let the end-users manage their identities as per their consent. However, the proposed implementation of software wallet has significantly less interoperability among enterprise blockchain applications, making the solution very rigid and less adaptable.

Polge et al. [21] provided the detailed comparison of five major permissioned blockchain frameworks Fabric, Quorum, Ethereum, R3 Corda, and MultiChain. The comparison metrics are scalability, performance, privacy, and adoption criteria. It also explained the consensus protocol used in the Quorum blockchain called “QuorumChain,” which is declared fast compared to relative consensus protocols. The respective research described the tradeoffs between the mentioned blockchains and identified that the drawbacks and benefits of using each approach depend on the use case scenarios. The research paper concluded with the lessons and suggestions for industrial blockchain practitioners and researchers. It suggested that industrial practitioners should select 2-3 frameworks and first test them in their own environment/settings and keep track of the queue size that limits the latency in case of too many simultaneous transactions. Secondly, it suggested that the researchers must compare various frameworks in the same environment as there is a significant gap in the previous literature. It also directed future researchers to work on optimising the consensus algorithms to enhance the performance of the blockchain system. The research provided valuable information; however, the versions used in the experimental evaluation are old for some blockchain frameworks.

Table 1 provides a summarized overview of the reviewed literature related to permissioned blockchains.

### 2.2. Blockchain-Driven IoT

Permissioned blockchain provides the enterprise-grade blockchain-based solution with only authorized entities capable of joining the solution. There are several architectural limitations of stable permissioned blockchain Hyperledger Fabric, and the redesign of its architecture with flexible endorsement policies and pluggable modules is inevitable. One of the significant issues is that blockchain is the novel solution and numerous blockchain frameworks exist. It is complicated to choose the best suitable blockchain solution as per the use case need. For the technical applicability, there is a need for new avenues of multidisciplinary blockchain research. Several investigations compared the popular blockchain frameworks like Corda, Ethereum, and different Hyperledger flavours and recognized the best among them; the review of comparisons increased the knowledge about the significant differences among different blockchain solutions and helped in determining the appropriate framework for resource-constrained IoT devices networks.

**Figure 2:**
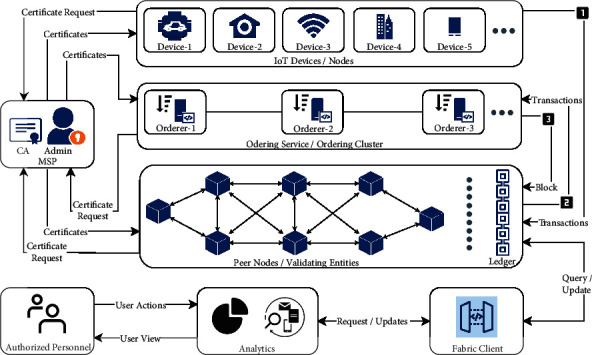
Blockchain-driven IoT architecture.

Public blockchain solutions offer numerous benefits but lack some of the most significant characteristics of standard industrial solutions. For industrial IoT-based network architecture, some requirements are lacking in public blockchains-like IoT devices or network participants needed to be identifiable, only authorized or permissioned entities must be allowed to become part of the system, high-transaction throughput performance requirement, low-latency transaction confirmation requirement, and confidentiality of the private transactions and data. Considering enterprise or industrial needs, the permissioned or private blockchain offers tremendous benefits without compromising the industrial needs and core utilities provided by the traditional blockchain. A generic IoT-enabled enterprise blockchain architecture based on Hyperledger Fabric is shown in Figure 2. With the help of membership service provider (MSP) and certificate authority (CA), the administrator enrolls entities-like orderer, IoT devices, peer nodes, and generate identity certificates for them. Peers or peer nodes are resource-rich nodes that host ledgers; smart contracts and endorsement of chaincode execution are the responsibility of the peer. Ordering nodes perform the transaction ordering via receiving the number of endorsed transactions from the peers, then a block is formed, and the number of orderer nodes forms an ordering service. The orderer initiates the network; IoT devices could perform transactions and store transactional data to the ledger once the network is started. When IoT devices interact and generate transactions containing data or information, the transaction proposal is sent to the peers for validation and endorsement as per the chaincode. After successfully validating the transaction and signing it, the peer sends it back to the ordering service; this step is known as a broadcasting endorsement.

When the orderer receives the transactions, it waits until the block or batch time to accumulate several transactions to constitute a block. It sends that group of transactions in the form of the block back to the peer for permanent storage. All the infrastructure is shared among different channels; however, only the peers who are part of the respective channel are involved in the whole process and ultimately store the blocks concerning their channel. The ordering service and CA are the guardians of the whole network; forging either will question the legitimacy of the whole network. The fundamental assumption in the scenario of blockchain-driven IoT scenario is that CA and ordering service both are trusted and secure; hence the network could trust the identities generated by them.

### 2.3. Consensus Methods in Blockchain-Driven IoT

Christidis and Devetsikiotis [22] provide the supply chain model implemented with blockchain. The proposed model maintains the delivery log for the shipment of containers via the secure blockchain storage mechanism. All the supply chain network entities could review the shipped containers in a cryptographically verifiable manner and trace them to reduce delivery delays; moreover, the mismanaged container item could be detected accurately. The issues that must be considered while deploying IoT solutions over blockchain-like transactional privacy, scalability, and network overhead were also discussed. However, the proposed model adopted a vulnerable security risks approach of canceling consensus mechanism via a whitelist scheme, which raises severe system security risks.

Yeow et al. [23] presented a thorough survey on the consensus methods used in blockchain and provided the thematic taxonomy of consensus mechanisms based on security parameters. The paper discussed the pros and cons of existing decentralized approaches and the commonalities and variances in consensus methods of blockchain networks. Moreover, it discussed the state-of-the-art consensus algorithms and their applicability in edge-centric IoT nodes networks. Finally, the study concluded by putting insights into research challenges and open issues related to IoT blockchain implementation and highlighting the deficiencies in centralized systems without blockchain. However, the paper discussed very little regarding the consensus mechanisms for permissioned blockchain and insight more regarding the consensus mechanisms in public blockchain solutions for edge-centric IoT nodes networks.

Dorri et al. [24] insight on the smart home case study for IoT and proposed a framework to induce traceability, security, integrity, and confidentiality. The proposed framework equipped smart homes with miners that induce high availability characteristics and computationally resource-intensive capabilities. Miners are responsible for internal and external communication handling. Each miner has its own preserved ledger used for auditing and controlling all in between communication. The proposed mechanism made the consensus mechanism light by eliminating the proof of work consensus method, which improved the speed of transaction and efficiency but then the system is more vulnerable to security risks.

Cao et al. [25] briefed about the use of blockchain in IoT devices networks and explained the fundamentals behind the popular consensus methods like proof of work (PoW), proof of stake (PoS), and directed acyclic graph (DAG). The paper also discussed the applicability of these popular consensus algorithms in IoT devices networks. The characteristics of the Tangle and Hash graph, which are based on the DAG consensus mechanism, are also discussed. The challenges in DAG-based consensus algorithms were also discussed and analyzed based on their applicability in IoT nodes networks. However, this paper provided significantly fewer directions about how the DAG-based algorithms can be made suitable for IoT devices networks with computational and communication constraints.

Huang et al. [26] proposed a credit-based security mechanism for blockchain-enabled IoT networks of industrial factories. The proposed mechanism used directed acyclic graph (DAG) based blockchain instead of traditional blockchain, which is more friendly for resource-constrained IoT devices in terms of storage and power consumption. Traditional chain-structured blockchain uses synchronous proof of work consensus mechanism, whereas the proposed scheme used an asynchronous credit-based consensus mechanism. The mechanism works by increasing computational workload extensively for malicious nodes whereas decreasing the same workload for honest nodes. However, due to the asynchronous nature of the consensus mechanism, it is vulnerable to security threats in some scenarios.

Khalid et al. [27] signified the importance of IoT as an emerging technology that is composed of heterogeneous devices that are connected via Internet. As IoT devices generate a huge amount of confidential and security-sensitive data, there is a need for efficient and reliable authentication mechanisms. The research proposed a lightweight decentralized authentication mechanism for the heterogeneous nature of IoT devices. The proposed mechanism is based on concepts of the public blockchain and fog computing. The proposed mechanism results are more reliable than state-of-the-art authentication techniques; however, the paper did not provide insights into the authentication mechanisms used in permissioned blockchain.

Biswas et al. [28] addressed the security and access control challenges solution with blockchain implementation in heterogeneous nature of the Internet of Things (IoT) networks. The core consensus algorithms used in enterprise blockchain are less suitable to scale for many IoT devices, and to scale them, the security of the consensus methods is to be downgraded. The research proposed a lightweight consensus mechanism for business blockchain as proof of block and trade (PoBT), which efficiently reduces block validation and computation time; however, the proposed mechanism is only valid for business blockchain and is not suitable for a public blockchain.

Raghav et al. [29] described the drawbacks of using blockchain technology in IoT devices network as high energy consumption, massive computation requirement, and high latency of blockchain-based consensus algorithms. Concerning the respective issues, a lightweight trust-free probabilistic consensus algorithm proof of elapsed work and luck (PoEWAL) is developed for IoT devices network environment that consumes less energy and has low latency. Experimental results demonstrate the low energy consumption, network latency, consensus time compared with the state-of-the-art consensus mechanisms at various difficulty levels; however, the paper lacks the significant experimentation to analyze in-depth resistance and security against cyberattacks.

Wang et al. [30] leveraged the lightweight blockchain and consensus algorithm to enhance the security of routing of swarm unmanned aircraft systems (UAS) networking. The respective investigation compared the proof of work and proof of stake in estimating the traffic status of UAS in swarm UAS networking and proposed the proof of traffic (PoT). PoT is a novel lightweight consensus algorithm that synchronizes lightweight blockchain blocks while staying in the limited power-constrained resources for UAS. The evaluation resulted in decreasing the routing consumption processes via lightweight consensus algorithm and lightweight blockchain. The paper optimized the performance and efficiency of resource-constrained UAS networks; however, the study lacks in-depth discussions on vulnerabilities and attacks on the proposed system.

Li et al. [31] signified the importance of blockchain-based lightweight consensus mechanisms in providing a secure, efficient, and reliable solution for resource-constrained IoT devices network. The paper proposed a lightweight modified PBFT consensus algorithm based on punishment penalty and rewards strategy that can reduce blockchain storage cost, delay of the consensus, and communication resources required by consensus. In storage systems, Reed-Solomon (RS) erasure codes are used to find and correct the erroneous symbols at unknown random locations. To optimize the blockchain solution, a scheme based on RS erasure code is introduced to reduce the overhead of recoverability of blockchain. However, the investigation lacks in determining the effects of using RS erasure code technology for storage optimization as it has high resource consumption, which is constrained in IoT devices network.

Frikha et al. [32] discussed the implementation of blockchain consensus algorithm in supply chain embedded architecture. This study shows that in the supply chain of distributed robotic systems running on embedded IoT hardware, the power consumption, and execution time are more significant challenges in addition to data security issues and the number of nodes scalability. The study proposed the software and hardware hybrid architecture of the proof of work (PoW) consensus algorithm with validation on the Ethereum blockchain which shows three times improvement in execution time and more than two times saving in power consumption. The respective results made the solution highly applicable in the supply chain case scenario; however, the variation of the PoW consensus algorithm is used which is less efficient in terms of power-constrained devices. In addition, the enterprise-grade need for organizational private data is also neglected via validation of the solution on the public Ethereum blockchain.

Zhang et al. [33] described that blockchain traditional consensus protocols have complications like high power consumption and token dependence which is not suitable for large cloud manufacturing systems. In response, this paper developed a proof of service power (PoSP) consensus algorithm for cloud manufacturing which calculates the total power of member nodes and optimizes the performance and power consumption of PoW. The experimental results show that the proposed consensus algorithm has better performance in terms of power consumption and transaction per second (TPS) throughput which makes it cloud manufacturing friendly for supply chain IoT systems. Moreover, this study also provided insights on novel ideas of cloud manufacturing management and trust evaluation in blockchain applications; however, this paper did not discuss the enterprise need for cloud manufacturing management concerning the private data needs of organizations.

Table 2 provides a comparison based review of different consensus mechanisms for IoT and determined their limitations.

In this section, we presented the literature related to blockchain-based IoT, signifying why or why not public blockchains are suitable for IoT. Public blockchains are best suitable for environments that involve public use of IoT devices in an open fashion. However, industrial IoT does not need everything to be opened and accessible by everyone.

## 3. Blockchain-Enabled Supply Chain System Design for IOT

This work aims to analyze different consensus methods in blockchain and determine their applicability in IoT resource-constrained devices networks. For this purpose, the blockchain-based supply chain case study is used to analyze consensus in IoT devices. Significantly known proof of work (PoW) consensus algorithm is a cryptographically secure proof. The prover proves that a certain amount of heavy computational puzzle is solved by finding the nonce value. The verifier nodes could verify the nonce value proof with minimal computation effort. PoW guarantees that the prover node has made the required effort to create the block of transactions for the blockchain; however, the heavy computation requirement for the prover node in PoW makes it unsuitable for IoT resource-constrained devices. Proof of capacity (PoC) is similar to PoW, but instead of relying on the computation power of nodes, it relies on the storage capacity of nodes. The more the capacity of the nodes, the vast dataset (plot files) it could hold, and then it could find the solution more quickly. However, the storage capacity of IoT resource-constrained devices is also not great to support proof of capacity consensus mechanism.

**Figure 3:**
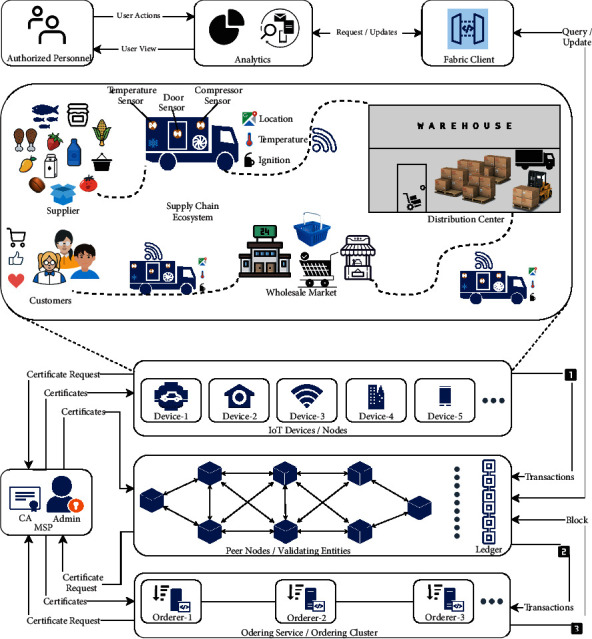
Blockchain-enabled supply chain system design.

Proof of stake (PoS) is among the most used consensus algorithm after PoW, in which a bidding system is introduced for nodes. When they bid, their specific stake is locked; if other nodes validate their solution, the stake is returned; otherwise, the network holds the winning node stake. PoS is energy efficient; however, its integrity is challenged by a “nothing at stake” problem. In “nothing at stake” problem, an attacker sends his transaction in fork-2, confirming it and then utilizing its computational resources to assist fork-1, to get the fork-1 win and fork-2 vanish that prone to the double-spend problem. There are solutions for this problem, but even if this problem is solved, this consensus method is not suitable for IoT networks as it involved a monetary stake that does not exist in IoT networks. This section describes the methodology of the improved mRAFT consensus algorithm in terms of latency and throughput and then plugging its module in a supply chain-based IoT network. The comprehensive design of the generic IoT-based supply chain management solution on top of permissioned blockchain Hyperledger Fabric is shown in Figure 3.

### 3.1. Supplier Module

In a hypothetical scenario, the supply chain ecosystem comprises of suppliers that produce any sort of product like electronic components, mined stones, seeds, fish or agricultural products, and many more. The aim of securing the whole ecosystem and ensuring transparency among the processes is to deliver the product to the end-user in a timely, secure, transparent, reliable, and reputable state. The product from the supplier is transited by using some transport medium that has embedded sensors like temperature sensors, compressor sensors, GPS tracking sensors, or any other sensor as per the use case requirements, depending on the nature of the product being transported. The rationale behind embedding the facility with sensors is to deliver everything in a controlled and monitored manner. For instance, as we know, the temperature must be under bounds for perishable products, so temperature sensors will help regulate temperature. In disaster cases, it will let the audit committee check that high temperatures could be the cause of food wastage; this will ensure transparency in the delivery process. All the data will be recorded in each step of the pipeline, and that data is to be stored on the blockchain, the mechanism of which will be described later. The bulk of the product is delivered from the supplier to the distribution center or warehouse in a controlled fashion. It could be observed that even this small-scale digitization of the individual stage of the supply chain management process could be very helpful in determining the inefficiencies along the transport process.

#### 3.1.1. Distribution Center Module

The delivered products have now reached the distribution center. However, it has a long way to travel to the final destination, the retail market where customers could buy the product. They know the product they are paying for is a quality product and every entity in the whole supply chain management process has track and proof of its legitimacy and quality standard. From the distribution center, now products have to be delivered to the wholesale market. The exact mechanism will be adopted as it was adopted from supplier to warehouse that is the transportation of the product in a controlled and monitored environment. In the described hypothetical scenario, the generic form of the supply chain management process is being discussed; however, there is a difference among supply chain management processes of different products. Lastly, the product is delivered from the wholesale market to the retail market for selling to customers in the same controlled and monitored fashion. The intelligent supply chain management workflow and storage of information over blockchain ledger provide cryptographic security that allows suppliers to undergo supply chain operations based on their unique identity. On the other hand, customers can view profiles of their concerned distributors to get the details of the originality of the product and the respective timelines of all the activities from the supplier of the product to the destination of the customer. Recording this information and IoT device transactions onto the blockchain allows immutable storage of all the events that have taken place in the long way of supplier, distributor, and wholesaler, which is also excellent for supporting auditing capabilities around the whole ecosystem.

#### 3.1.2. Blockchain Data Storage Module

The storage of transactions between IoT devices on the blockchain is based on the secure and immutable smart supply chain management solution. The core architecture and infrastructure of the proposed solution will be discussed onwards. Before describing the respective research work on the consensus method across blockchain distributed nodes, it is worth explaining the internal transaction flow and architecture of an enterprise-grade blockchain Hyperledger Fabric with a supply chain management-based case study. The proposed solution in this research is implementable with other blockchain frameworks; however, choosing Hyperledger Fabric is because of its characteristics of being open source, flexible, and modular architecture; moreover, it is the most stable frameworks among all permissioned blockchains. The transactional mechanics in a standard transaction execution between supply chain IoT devices will be discussed now. As in permissioned blockchains, this is the responsibility of the Membership Service Provider (MSP) to handle the task of approving only permissioned participants to join the network. In cooperation with the Administrator and Certificate Authority (CA) as its submodule, MSP manages the supply chain IoT device IDs and authenticates them to use the network. The administrator will create the channel between the related IoT nodes inside the respective unit of the supply chain ecosystem that allows them to transact confidentially as only concerned entities must be allowed to access their related information. IoT devices register and enroll themselves with the CA of their respective unit and, in return, receive the required cryptographic material that they could use to authenticate themselves to the network. The smart contract of the Fabric that is chaincode representing the initial state of the ledger is installed on peers and deployed to the channel. Chaincode contains the endorsement policies that infer the terms and conditions for legitimate transactions over the network.

**Figure 4:**
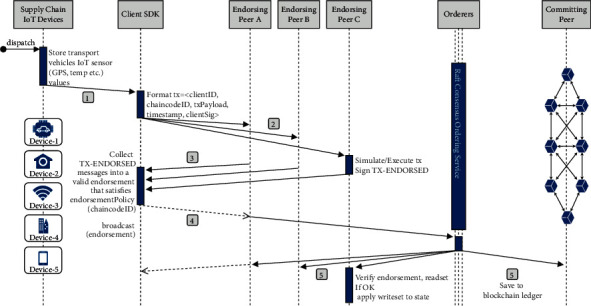
Transaction execution flow.

All the interaction between the supply chain-based ecosystem is done via the connected peer; through that peer, they will send the transactions and interact with the ledger. As shown in Figure 4, consider a supply chain-based scenario in which the transport information related to the supply chain products like temperature, GPS location, compressor sensor value is to be recorded on the blockchain as a piece of exchange information. During shipment at a time instant, the transport information is stored to the ledger; for that, a request is generated to the connected peer node. According to the endorsement policy, the peers connected to the respective unit must endorse the transaction before further proceeding. Before sending the request to a peer, an application leveraging supported SDK uses available APIs to generate a transaction proposal. The transaction proposal is a complete request to invoke a chaincode method with related input parameters, the aim behind that is to read or update the state of the ledger. SDK, in return, takes the request and binds it in the required format along with signatures (cryptographic credentials) of the IoT devices such that peers could verify that transaction is from a legitimate source. After receiving the transaction proposal, the endorsing peers verify it as per the required standards. It is verified that the transaction proposal is well-formed, it is not resubmitted to the peer, the signature is valid, and the respective source is authorized to perform that operation (as per the writing policy of the channel).Transaction proposal resubmission is a form of replay-attack in which an exact copy of the previous transaction is again submitted to the peer that could wrongly modify the state of the ledger. Chaincode in the described case is about storing the IoT sensor device values to the blockchain ledger; upon successful verification of the required standards, the peer executes the chaincode with the input parameters on the current state of the ledger. It is important to note here that the execution result is response value, write set, and read set only; however, no changes are committed to the ledger at this point. The resultant values and the endorsement peer signatures are sent back to the SDK as transaction proposal response. Suppose the transaction proposal is only querying the ledger. In that case, the response is displayed to the user. There is no need to submit the transaction response to the ordering service; however, there is a need to update the ledger in our case. For that, the transaction response is submitted from SDK to the ordering service to update the ledger after inspecting that the required number of peers endorsed the transaction or not. Fabric SDK broadcasts the IoT device signatures, endorsing peer signatures, read/write sets, and channel ID to the ordering service. The ordering service receives transactions from all the channels in the network, orders them chronologically, and creates blocks of transactions for each channel. Blocks of transactions are delivered to all peers, including the endorsing peer, and it is verified that there have been no changes in the ledger since the transaction execution by the endorsing peer. Upon receiving blocks, each peer appends the blocks to the respective chain of the channel, and for each valid transaction, the write sets are committed to the current state of the blockchain ledger.

### 3.2. Raft Consensus Execution Overview

Many distributed public blockchains like Ethereum and Bitcoin use probabilistic consensus algorithms to agree on the single state of the ledger. This consensus guarantees the ledger consistency with a high level of probability; however, they are still vulnerable to forks in which different network participants have different views about the state of the ledger. In Hyperledger Fabric, everything works in a deterministic fashion in which orderers working in collaboration with other orderers form an ordering service responsible for doing transaction ordering. The consensus mechanism used inside the ordering service is deterministic; there are no forks like permissionless blockchain networks. In Fabric, endorsement of chaincode execution is isolated from the ordering of transactions inside the ordering service that provides Fabric an edge over other blockchain networks in terms of scalability and performance; moreover, it hinders the bottlenecks that occur when the same nodes perform ordering and execution. As discussed in the previous section, each peer belongs to an organization; similarly, each orderer belongs to the organization and uses the certificate issued by the CA for the organization. IoT client SDK receives corresponding transactions endorsed by the endorsing peers, respectively, and sends each transaction to orderers, ordering service upon receiving numerous transactions package them together into block. The order of transactions must be agreed upon between the orderers in an ordering service through consensus. It is also the responsibility of the ordering node to distribute the packaged blocks to peers, and then peers apply these blocks consistently to the ledger over the blockchain network.

**Figure 5:**
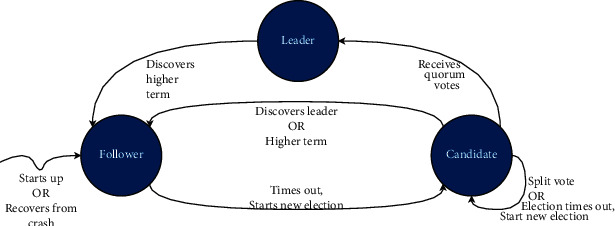
State machine model for the Raft consensus algorithm.

Now diving deep into the mechanism of orderers for reaching the strict singleton order of the transactions inside an ordering service. In Fabric, the consensus mechanism to achieve this is based on Raft protocol [34]. Raft is a crash fault-tolerant (CFT) consensus algorithm that represents the leader and follower model in which inside an ordering service, a leader is elected per channel, and all the followers replicate its decisions. Raft consensus protocol keeps a replicated log, an append-only data structure. The leader is responsible for adding new entries to the log and asking its followers to replicate it. Every coming request is forwarded to the leader in charge of the replicated log, and a response is delivered to the IoT client SDK upon storing the data safely. Raft consensus protocol comprises the leader selection phase, log replication phase, and safety principles to ensure log safety. There are three states possible for every node in Raft: “leader,” “follower,” and “candidate” state. Upon startup of the system or a node recovering from the crash, the node is in a “follower” state. Each node has a random timeout; the node that times out first converges to the “candidate” state and starts the selection process. Quorum is the term used in Raft to represent the majority of the nodes in the Raft network. Upon receiving votes from the quorum of nodes which means majority of nodes, the candidate node converges to the “leader” state and sends heartbeat messages to its followers. Empty append entry messages are considered as heartbeat messages and are periodically sent to the followers so they could be alerted about the liveliness of the leader. Subsequently, in case of a leader crash, a random timeout on some follower node will be triggered, and the node will converge to the “candidate” state and start a similar selection process. Suppose no leader could receive the votes from the quorum nodes and selection times out or two nodes timeout at the same instant, which would result in a split vote. In that case, the selection process is repeated until the new leader is elected successfully. While in a “candidate” state, the candidate receives a heartbeat message and discovers a leader with a higher term; it will step down immediately and converge to the “follower” state. In Raft consensus, all transactions pass through the leader node that causes congestion at the leader side; to mitigate the leader bottleneck overhead is a significant research challenge in Raft consensus [35]. There is no logical time in Raft; in fact, time is based on a sequential counter called a Term that is the period from the start of a selection process to the instant when a node does not receive a heartbeat from the leader, times out, and starts a new selection. Nodes in Raft consensus protocol transit from one state to another as shown in Figure 5, in a particular term, at most only one leader could be elected that guarantees selection safety.

### 3.3. Modified RAFT (mRAFT) Consensus Protocol

Raft undergoing all operations and proceeding requests through the leader has a high probability of becoming a bottleneck point. So, in the proposed mRAFT consensus protocol, instead of loading all the transactions through the single leader, the follower nodes also aid in disseminating the information over the network that would reduce workload from the leader and make it available for fulfilling the IoT client SDK requests.  Table 3 describes the different modules of the Raft algorithm and maps their improved mRAFT versions and their explanation that helps in increasing the efficiency of the overall consensus process between orderers in an ordering service and ultimately reduce the transaction latency of the resource constrained IoT nodes and help them scale at large.

#### 3.3.1. Leader Selection Phase

In Algorithm 1, in the blockchain network initialization phase of the Raft protocol, the node initializes the variables; presentTerm represents the term in which node is operating, votedForNode represents the candidate node for which the node has voted for, committedLength represents the length of immutable log records that are committed and log data structure represents the mapping of log entries with their corresponding term. All these variables are stored on the local storage that in node crash scenario will let the node recover from its last active state of the log. Other variables initialized at the start are stored in volatile memory, and their contents will be lost if a node crashes. When a node recovers from the crash, it sets the variables in its volatile memory to default values, and the default state will be the “follower” state. Now coming to the scenario when a node did not receive a heartbeat from the leader node, it will suspect the leader node has failed or if one candidate node selection times out and it is unsuccessful in receiving quorum votes, the selection process starts. With the selection process, the node increments the presentTerm by 1, changes its state to the “candidate” state, votes for itself, adds its nodeId to the votesReceived set of nodes. If a log is empty then the recentTerm will be initialized to 0; otherwise, it will be set to the corresponding term of the last entry in the log. After that, the candidate node packs all the required information and has to send the VoteRequestMsg message to all the follower nodes, due to which this instant could become a bottleneck for the candidate node for disseminating the VoteRequestMsg message to a large number of follower nodes. In the improved version mRAFT proposed by this research, instead of the candidate node sending the VoteRequestMsg messages to all the nodes itself, it only selects *k* random nodes and sends them the message. Upon receiving the message, it will be the responsibility of the receiving follower nodes to disseminate the message to further *k* random nodes that will follow the same message sending mechanism again and ultimately, the message is delivered to the whole Raft network. The purpose of this broadcast is to reduce the load from the candidate node via triggering the follower nodes, which will share that load and in result, reduce the latency of the VoteRequestMsg message efficiently. As soon as the candidate node sends the VoteRequestMsg message to the *k* random nodes, it starts the selection timer, and the selection must be completed in the selection timeout; otherwise, the selection process will be repeated as stated earlier.

In Algorithm 2, when a follower node receives VoteRequestMsg message from the candidate it first checks that its local log is consistent with the candidate node or not, it will let the follower node decide on whether to vote for the candidate or reject the request. To check log consistency, the follower node compares the term of the last entry in the log with the term of the last entry in the log of the candidate that is passed as an argument in the VoteRequestMsg message. The log of the candidate is considered good if the term of the last entry in the candidates' log is greater than or equal to the term on the last entry in the followers' log and log length of the candidate is greater. It is also possible that outdated VoteRequestMsg from the old term is received by the follower, due to the fact on the follower node it checks if the term is greater or equal to the candidate term or not. If duplicate VoteRequestMsg with the same term is received by the follower then votedForNode must be for the same candidate or null. If logOk and termOk are true then the follower node updates its term to the candidates' term, its current state to the “follower” state updates its votedForNode variable to the candidate ID for which it is voting for. The follower node then disseminates the VoteRequestMsg message to the *k* random follower nodes and sends the VoteResponseMsg back to the candidate as true. If logOk and termOk are false then the follower node sends back the rejection VoteResponseMsg as false to the candidate.

#### 3.3.2. Log Replication Phase

In Algorithm 3, candidate upon receiving VoteResponseMsg compares the current term of the follower node that is passed in arguments with its presentTerm. The desired case is when candidates' term is equivalent to followers' term and VoteResponseMsg is granted true, in that case, the candidate will add ID of the follower node as voterNodeId to the votesReceived list; this operation will be an idempotent operation that means duplicate VoteResponseMsg from the same follower will not increase the votesReceived count. With each VoteResponseMsg as true, candidate also checks if the number of votesReceived is equal to the quorum nodes, in that case, the candidate will win the selection and transit to “leader” state and cancels the selection timer. The candidate will then update the variables for the follower nodes, sentEntriesLength that represents the number of entries already sent to the follower node from the beginning of the log, and ackedEntriesLength that represents the number till which acknowledgments for log have been already received from the follower node and then the elected leader disseminates the log entries to the *k* random nodes. ReplicateLogMsg function is called with arguments as nodeId that represents the leaderNodeId, follower that represents the node to which the message is being sent and sentEntriesLength that represents the list of the count of the entries already sent to each of the follower. The reason to pass sentEntriesLength as an argument to the ReplicateLogMsg function is to make the receiver follower nodes further disseminate the log entries to the *k* random follower nodes. The main aim of calling the ReplicateLogMsg function is to alert the followers about the presence of the new leader in the system.

In Algorithm 4, when a write request is received at the node, it checks if it is the “leader” state. If not, then it forwards that request to the current leader node. Upon receiving the broadcast message, the leader node appends the record to the log that includes the message and the current term. The message is only appended at this stage to the log as a new log entry. This message will be committed once the leader sends that append entry request to the follower nodes and receives acknowledgments from quorum nodes. Leader upon appending the log with the new entry updates its ackedEntriesLength to the new length of the log and selects *k* random nodes and calls the ReplicateLogMsg function for them so their log is also updated till the latest entry and then those followers disseminate the broadcast message with ReplicateLogMsg function to further *k* random nodes. In parallel, in the background, the leader node keeps on calling the ReplicateLogMsg function periodically for each of the follower nodes, so they might get an update if some messages get lost or sometimes for the commit purposes. The ReplicateLogMsg function, which does not necessarily always contain the new entries, is also called to send heartbeats to each of the follower nodes so they could get an alert of the liveliness of the leader node. The definition of the ReplicateLogMsg function is unchanged in the proposed research; however, an additional parameter as sentEntriesLength is added that has to be passed as an argument so when the follower node disseminates the message received by the leader node, it could access the sentEntriesLength of each of the follower nodes to which it is sending the message. ReplicateLogMsg function is called on the leader node when there is a new message entry in the log or the leader also calls it with empty entries for sending periodic heartbeat messages to each of the follower nodes to alert them that the leader is still alive. In ReplicateLogMsg function, information related to the number of entries that are already sent to each of the follower nodes is taken from sentEntriesLength and then entries variable is assigned the suffix of the log entries ahead from sentEntriesLength. In the next step, the variable prevlogRecentTerm is assigned the value of the term in the last entry of the log; this variable will be used afterward for consistency check at the follower node. After that, the LogRequestMsg message is packed with a whole bunch of information related to log characteristics as well as the new entries and is sent to the follower node that the follower node will use to update its log.

In Algorithm 5, when the LogRequestMsg message to update log is received at the follower node from the ReplicateLogMsg function; it first compares the argument variable term with its presentTerm if the argument passed value is greater, then it updates its term to the latest term, resets its votedForNode variable to null, converges to “follower” state, and updates its presentLeader variable with the new leader. Then, follower node will then do a consistency check of its log so that it could decide either it had to accept those log entries or ask the leader for more entries from the history to make its log consistent with the log of the leader and fill the gap in its log. If the term of the follower node is the same as on the leader node and log is consistent then the follower is in the situation to update its log with the new entries. To add the new entries to the log of the follower, the AppendEntriesMsg function is called with the new entries and required information. After calling AppendEntriesMsg function as now, the follower has received the new entries hence its ack variable that represents the number of entries received by the respective follower is updated. The new entries are then disseminated through ReplicateLogMsg function to the *k* random follower nodes as per the sentEntriesLength of each follower so the follower node could also update its log with the new entries and disseminate them to further *k* random nodes. The LogResponseMsg message with true result is packed with all the required information and is sent as an acknowledgment to the leader node as a success response. On the other hand, if the log of the follower is not consistent with that of the leader then LogResponseMsg with the false result is sent to the leader so that the leader node sends back some previous entries from its log and then the follower node could make its log consistent with the leader node.

The purpose of the AppendEntriesMsg function is to update the log of the follower node with the entries that have been received by the leader node. For that first log consistency of the follower and the leader is checked by comparing the length of the logs, if the length of the log of the follower is greater and the term of last entry of log is nonequivalent, then it is obvious that it contains garbage entries hence its log is truncated and those entries are deleted. Raft guarantees that those discarded entries are not committed entries. On the other hand, if the log of the leader is greater than the log of the follower node, then the new entries are appended to the log sequentially in an idempotent manner so that if any entries are repeated, then they are not added again to the log. After that, if the leader has more committed entries than the follower node, then the new messages are delivered to the IoT client SDK, and committedLength of the follower is updated equivalent to the leader node. As the follower node returned the LogResponseMsg acknowledgment message to the leader, now the leader has to receive those messages and take action. If the term in the LogResponseMsg message from the follower node is lower than the term of the leader node, then it will ignore the message as it is outdated. Contrary to that, if the term in the LogResponseMsg message from the follower node is greater than the term of the leader node, then that means there is a new leader in the system; hence the old leader steps down, resets its votedForNode variable, and converges to “follower” state. If terms are equivalent and LogResponseMsg is true then that means the follower has accepted those log entries and now acknowledging the receipts hence sentEntriesLength that represents the entries sent to the follower node and ackedEntriesLength that represents the entries acknowledged by the follower node both are updated. After that, CommitLogEntries function is called that checks for the acknowledgments from the quorum nodes and commits the entries to the log. If terms are equivalent and LogResponseMsg is false then that means the follower has some missing entries hence the leader decrements the sentEntriesLength variable for that follower and calls ReplicateLogMsg function with one old log entry included, this could happen multiple times until the log of the follower node becomes consistent with the leader node.

A leader can commit the log entry once the quorum of nodes has acknowledged it, as it is then safe to commit that message afterward. When an entry is committed to the log, its message is delivered to the IoT client SDK, which means that the request is successful. CommitLogEntries function checks for the length of the log till which the acknowledgments have been received from the quorum of the nodes. Suppose that acknowledged length of the log entries is greater than the already committed log length, and the log is consistent as per the current term. In that case, each entry since the last committed entry is committed one by one, and the message is delivered to the IoT client SDK. After that, the committedLength is updated to the newly committed length of the log so when the next LogRequestMsg message will be sent by the leader to the followers, the value of this committedLength will be included that will tell the followers up to which point they could commit their entries of the log. In our mentioned IoT-based supply chain scenario, there are a lot of transactions between the IoT nodes. If the leader node directs all the operations, it will become the bottleneck point. However, the follower nodes are sitting idle most of the time that creates an imbalance in the workload of the peers. The proposed improvements in the Raft consensus mechanism for Hyperledger Fabric utilize the follower nodes in disseminating the VoteRequestMsg messages, AppendEntriesMsg message, and also the replication of the log messages via ReplicateLogMsg function among the followers. Follower nodes, upon receiving the messages, disseminate the messages to a further subset of nodes, due to which finally the message is propagated through the whole network.

## 4. Experimental Evaluation

### 4.1. System Configuration and Inputs

The system build and test environment are implemented using Linux virtual machine services. The Hyperledger Fabric network is built, run, and tested using the Raft and mRAFT consensus protocol. The environmental parameters for software and hardware are shown in Table 4. The prototype solution for experimental evaluation and testing is based on a virtual server hosted on a laptop computing machine with limited computational resources and capabilities. It must be kept in mind that in the production environment, the blockchain nodes will be held on server machines with heavy computational capabilities whose performance could differ in terms of scale. However, the rationale behind the results would be the same.

The blockchain performance benchmark tool used is the official Hyperledger Caliper tool that measures the performance of the various blockchain systems by using a set of predefined cases. Hyperledger Caliper uses a set of neutral and agreed upon rules to evaluate different blockchain solutions. In addition to a customizable test flow and workload, Caliper produces a report that contains performance indicators such as transaction success rate, transaction and read throughput, transaction and read latency, and resource utilization. In this study, Hyperledger Caliper is used to test, analyze, and compare the performance of the different configurations of the blockchain solution based on Raft consensus and modified mRAFT consensus mechanism. Caliper is a benchmark service that generates a workload against a specific System under test (SUT) and constantly monitors its execution responses in each cycle. After carefully monitoring the responses against a whole bunch of cycles, generate a comprehensive report of SUT responses. Hyperledger Caliper requires predefined inputs independent of the used system under test to run a benchmark.

**Figure 6:**
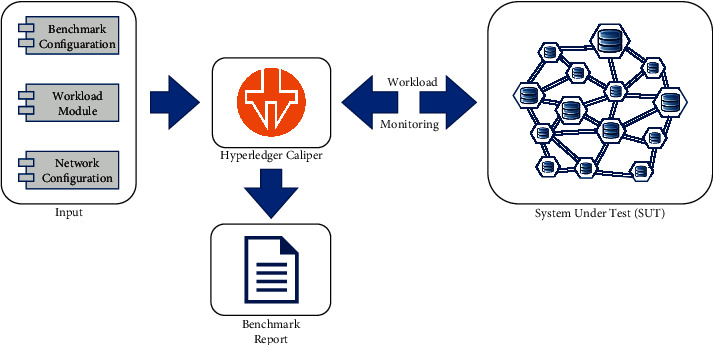
System benchmark testing environment.

As shown in Figure 6, these inputs include a Benchmark Configuration File that contains the information on how the benchmark should be executed. It also includes the rounds needed to be executed and the rate at which transactions should be submitted. This file is independent of the SUT; hence this file will remain the same for testing both the blockchain systems with Raft consensus and the system with mRAFT consensus. The second is the Network Configuration File which is partially SUT specific; this file also contains the topology of the SUT, the endpoint addresses of its nodes, a list of the identities and clients present in the network, and what smart contracts Caliper has to interact with. To maintain the standard configuration for both the systems under test, the network configuration files will be set the same to meet the benchmark standard except the topology configuration. The third input to the Caliper is workload modules that generate the content for the transaction when Caliper schedules transactions for a given round. Workload modules are set to default for both the systems under test.

### 4.2. Results and Discussion

**Figure 7:**
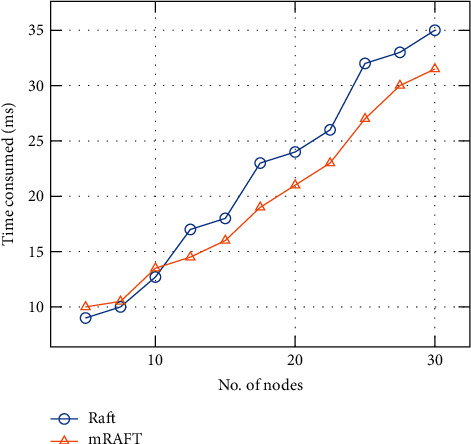
Leader selection time on varying number of nodes.

In the leader selection phase, as shown in Figure 7, the mRAFT consensus algorithm and the Raft consensus algorithm work the same on the small number of nodes as the dissemination of the vote request messages through the follower nodes has minimal effect on the small number of nodes till 11 number of nodes. However, it could be seen on greater than 12 nodes that on a large number of nodes in the Raft consensus protocol, the selection time is greater because of the bottleneck point for the leader to disseminate the vote request messages to the follower nodes. In this scenario, the mRAFT consensus protocol has less selection time, as discussed in Chapter 3. The idle follower nodes are being used to disseminate the messages to further random follower nodes, and no bottleneck occurs on the leader side. There is a linear increase in leader selection time after the 13 number of nodes on the Raft consensus algorithm. While increasing the number of nodes, the mRAFT consensus algorithm provides a more efficient leader selection time and inhibits the leader node from queuing and delaying the transactions.

**Figure 8:**
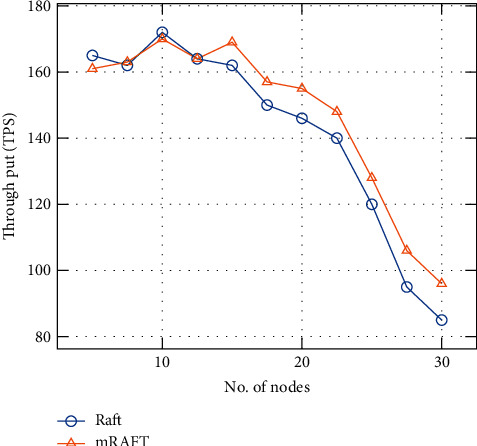
Throughput of the network on varying number of nodes.

In the log replication phase, the experiment is done for concurrent 350 transaction requests on a varying number of nodes to look for the comparative behavior of throughput for both the systems under test. As shown in Figure 8, it could be seen that the network throughput on the different number of nodes is almost the same for the smaller number of nodes for both the Raft consensus and mRAFT consensus algorithm. However, when the number of nodes crosses 13, the mRAFT consensus algorithm provides better throughput as the follower nodes are efficiently sharing the workload of the leader node. In return, the leader node utilizes the spare power in the validation of the remaining transactions. As a result of which the better throughput is delivered in the overall Hyperledger Fabric network. It can also be observed that the increasing number of nodes is inversely proportional to the throughput performance. Utilizing the follower nodes in disseminating the information also limits the capacity of the follower nodes for doing operations other than the Hyperledger Fabric ecosystem.

**Figure 9:**
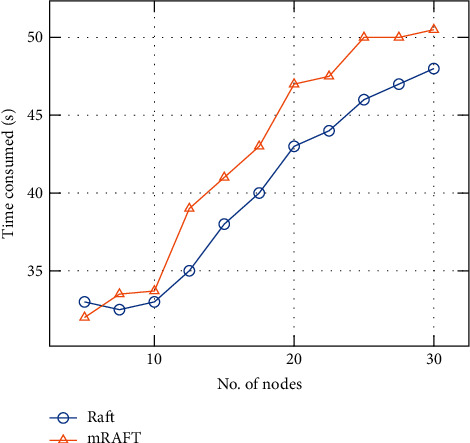
Network latency on 350 transactions and varying number of nodes.

Figure 9 shows the comparative latency on 350 sequential transaction requests on the Raft consensus algorithm and the mRAFT consensus algorithm on a varying number of nodes. This experiment aims to observe the behavior of the mRAFT consensus if transaction requests come in sequence one after the other. The sequential transaction injection means the new transaction always comes after the full completion of the previous transaction request. This experiment helps analyze the latency of the single transaction and determine how much time the algorithm is consuming to complete a transaction on a varying number of nodes. The results show that the latency for the sequential transactions is not fascinating as each log replication message in mRAFT consensus takes more time than the Raft consensus algorithm. More latency for sequential transactions in mRAFT is due to the mechanism introduced in mRAFT of utilizing follower nodes parallel to the leader node. However, when transactions are injected one after the completion of the previous, then the parallelism is of no use.

**Figure 10:**
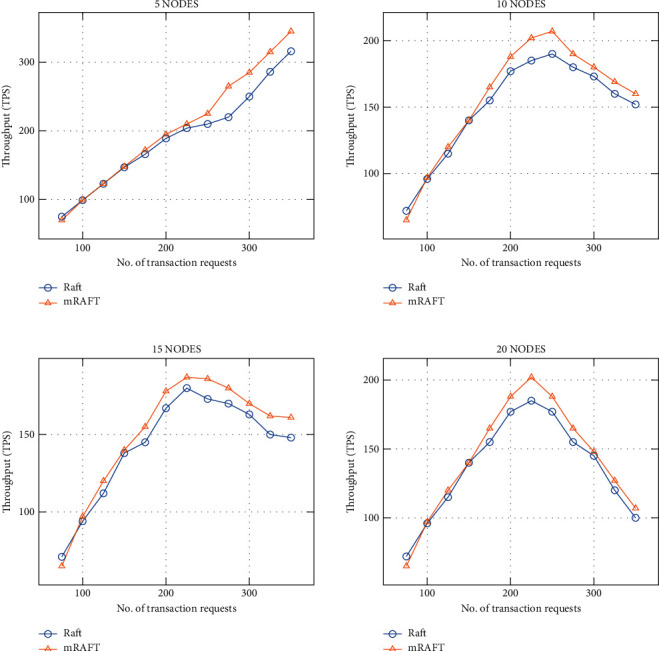
Throughput on concurrent transactions under 5-20 nodes.

The concurrent transactions throughput experiment shows the behavior of both the consensus algorithms when a varying number of transaction requests come at the same instant.  Figure 10 depicts the comparative throughput of the mRAFT consensus and Raft consensus algorithm upon receiving the concurrent transaction requests in a 5-20 nodes network. This experiment illustrates the throughput efficiency of both the systems under test if the leader node receives multiple concurrent transaction requests in Raft consensus. The results show that the successful transactions per second rate for mRAFT consensus are relatively higher than the Raft consensus algorithm because of the more efficient utilization of the leader and follower nodes. However, after reaching a threshold, the throughput rate of both the systems under test is leveled off due to the machine resource capacity. Similarly, it could be observed in 5, 10, 15, 20 number of nodes that the throughput is greater as per the injected transactions under a small number of nodes. However, with the increasing number of nodes, the throughput also reduces because of the large number of nodes participating in the consensus process.

**Figure 11:**
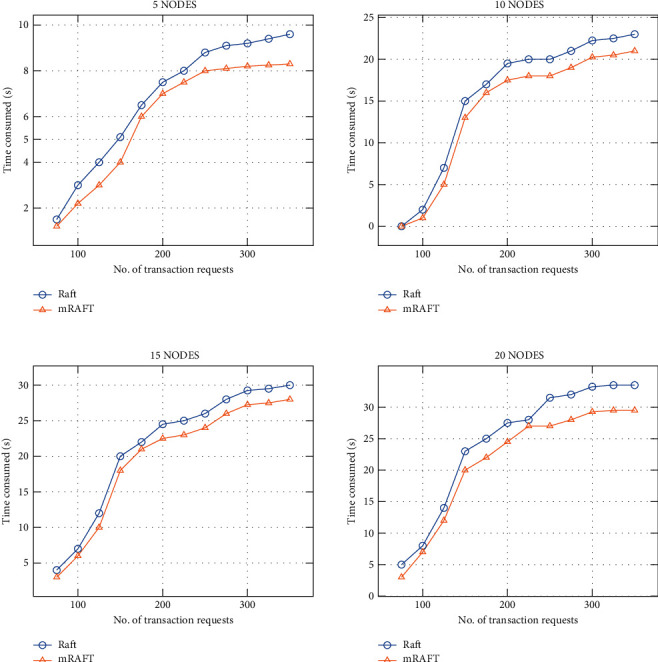
Network latency on concurrent transactions under 5-20 nodes.

As shown in Figure 11, it is significant to experimentally analyze the latency of the systems under test upon receiving the concurrent transaction requests at almost the same instant on a 5-20 fixed number of nodes. This depicts whether the system handles concurrent transactions efficiently or not. There is a confusing difference between Figures 9 and 11 as both show the latency results in experimentation. However, the former shows the latency when sequential transactions are received one after the completion of the previous, while the latter shows the latency for concurrent transaction requests. The result shows that the latency of the mRAFT consensus algorithm is better as compared to the Raft consensus algorithm when concurrent transaction requests are injected into the system because the system is now capable of executing transactions in parallel. Similarly, it could also be observed that the latency of the concurrent transactions increased with the increasing number of nodes.

The performance of the mRAFT consensus protocol is evaluated with the help of experiments. The parameters such as leader selection time, network latency, and throughput are considered to perform extensive experiments to determine the effectiveness of the mRAFT in the Hyperledger Fabric system. The experiment is conducted to test the efficiency of the mRAFT consensus and Raft consensus in leader selection time. The results indicate the improvement in the mRAFT consensus algorithm when the number of nodes is increased. Then the experiment is conducted to measure the throughput on a concurrent fixed number of transactions and a varying number of nodes. The respective experiment signified the superiority of the mRAFT consensus algorithm. Similarly, an experiment to measure network latency is conducted on a fixed number of sequential transactions. However, the individual transaction latency of mRAFT is not much different from the Raft consensus, which shows mRAFT is efficient when concurrent transactions are available. When there are concurrent transactions injected into the system, the other idle follower nodes share the hectic workload of the leader node and boost the efficiency of the system. In another experiment, throughput on varying concurrent transaction requests is observed under several fixed number of nodes. The results of the respective experiment demonstrate the efficiency of the mRAFT consensus algorithm compared to the Raft consensus. Finally, the experiment is conducted to test the network latency on varying concurrent transaction requests under several fixed number of nodes. The results of the respective experiment indicate the mRAFT consensus algorithm consumes less time in comparison to the Raft consensus; however, the concurrent transactions must be available in the transaction pool to get the required efficiency.

## 5. Conclusion and Future Work

The respective research work initially provided a detailed review of the literature and analyzed the impact of blockchain technology in solving scalability, transparency, and fault tolerance issues in enterprise IoT networks. The review helped in understanding the rationale behind blockchain and IoT and their core difference from traditional technologies in providing digital solutions. Moreover, the technical characteristics are analyzed for the most appropriate blockchain-based consensus algorithm concerning resource-constrained IoT devices networks. The literature related to the analysis of the applicability of different blockchain consensus mechanisms in IoT is reviewed to understand the suitable consensus mechanism for IoT better as the respective devices are resource-constrained in terms of storage and power consumption. There is also a concept reviewed in literature in which IoT devices resource-constrained nature smart homes are loaded with high-performance computing resources to provide additional resources for the computational tasks of IoT resource-constrained devices such that providing them aids in undergoing lightweight consensus among IoT devices. Moreover, the literature review revealed strategies that introduce rewards in consensus algorithms that ultimately proposed the evolution of credit-based consensus mechanisms that work by increasing the computational workload for malicious nodes while decreasing the workload for honest nodes. Analysis of the technical characteristics of IoT-focused consensus algorithms identified the best suitable parameters in designing consensus algorithms for IoT-based blockchain frameworks. This study also analyzes the consensus algorithms in specifically permissioned blockchains for their applicability in IoT devices networks and proposed improvements in the Raft consensus algorithm based on Hyperledger Fabric in the form of mRAFT consensus. The aim behind the improvements is to make blockchain adoption more convenient for resource-constrained IoT devices via improving the throughput and latency of the consensus algorithm. In our mentioned IoT-based supply chain scenario, there are a lot of transactions between the IoT nodes. If the leader node directs all the operations, it will become the bottleneck point. However, the follower nodes are sitting idle most of the time that creates an imbalance in the workload of the peers. The proposed improvements in the Raft consensus mechanism for Hyperledger Fabric utilize the follower nodes in disseminating the VoteRequestMsg messages, AppendEntriesMsg message and also the replication of the log messages via ReplicateLogMsg function among the followers. In the improved algorithm mRAFT, the workload from the leader node is reduced. In return, the leader efficiently utilizes the idle follower nodes in disseminating the vote request and log replication messages. The effectiveness of the improvements in the proposed algorithm is demonstrated with the help of a detailed experimental study. The results reveal that the proposed improvements in the Raft consensus protocol provide a better selection time, latency, and throughput. The follower nodes efficiently flood vote request messages for consensus in a larger number of nodes. Moreover, the mRAFT consensus provides significantly better network throughput on a varying number of nodes than the original Raft consensus for a larger than 13 number of nodes. Another experiment is done to analyze the latency in conducting 350 transactions on a varying number of nodes. The proposed mRAFT algorithm took more time when sequential transaction requests were injected, one after the completion of the other as individual transactions consume more time. The throughput of the algorithm is also improved on concurrent transaction requests under several fixed number of nodes. Ultimately, the experiment is conducted to analyze network latency on concurrent transaction requests under several fixed number of nodes, demonstrating the superiority of the proposed algorithm mRAFT. In general, the latency of the individual transaction is greater in the improved version mRAFT as the transaction requests are disseminated through the follower nodes. However, in a system with numerous transaction requests per unit of time, the algorithm provides better throughput, making it best useable in resource-constrained IoT devices networks. A number of considerations to improve the read and write operations of the Raft consensus algorithm for Hyperledger Fabric are also in the pipeline. For instance, the research challenges related to utilizing the quorum nodes in read or write transactions as their reliability is equivalent to the leader would further optimize the consensus process. Moreover, the consideration of non-occurrence of byzantine failures in the consensus process reduces the real-life applicability. There is room for research in making byzantine fault-tolerant consensus algorithms for permissioned blockchains.

## Figures and Tables

**Algorithm 1:**
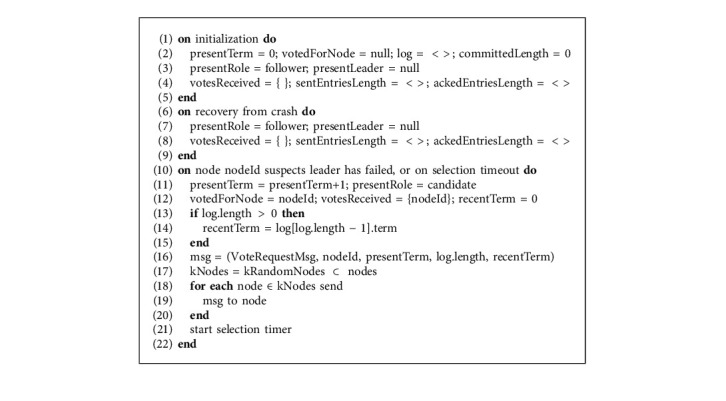
Blockchain network initialization.

**Algorithm 2:**
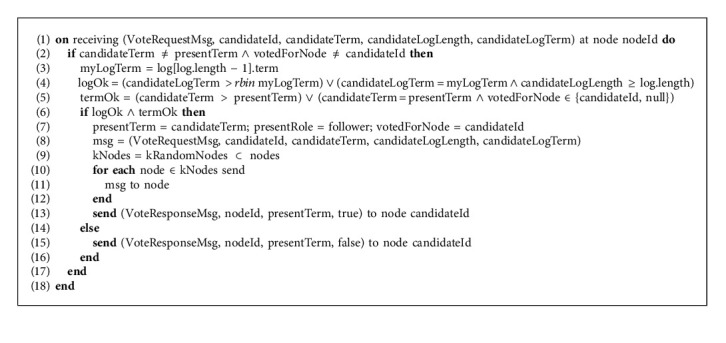
Voting on a new leader, at FOLLOWER side.

**Algorithm 3:**
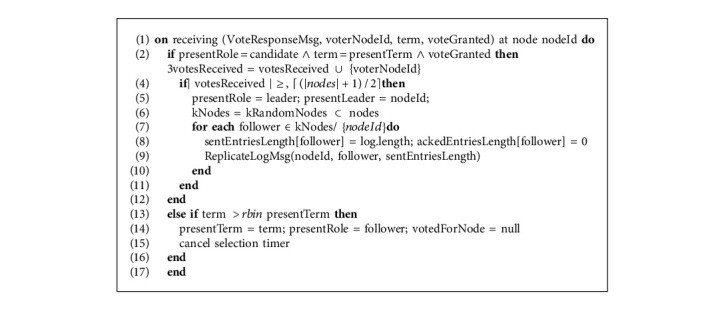
Collecting votes at CANDIDATE side.

**Algorithm 4:**
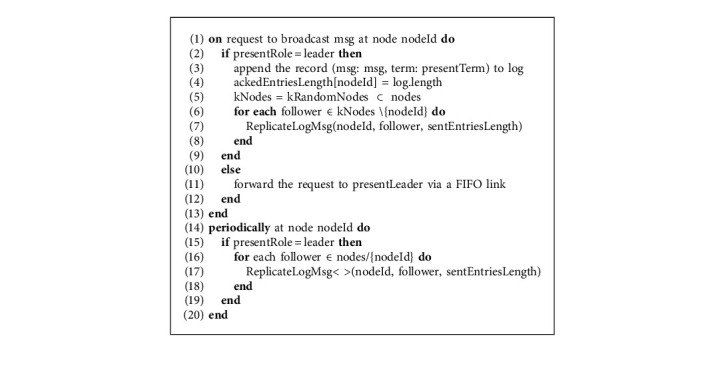
Broadcasting messages.

**Algorithm 5:**
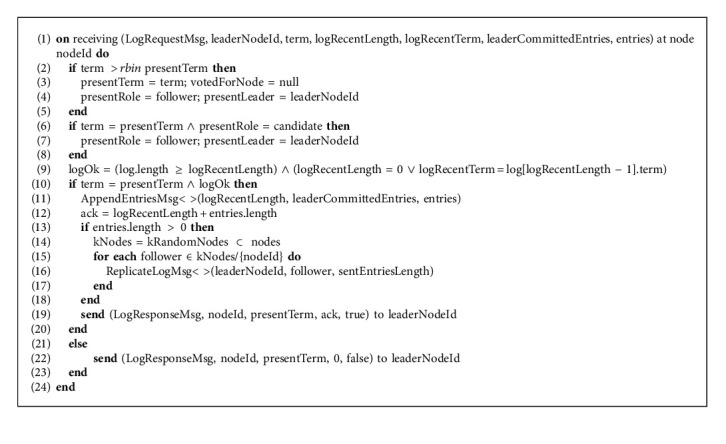
Followers receiving messages at FOLLOWER side.

**Table 1:** A review of permissioned blockchains.

Authors	Year	Approach	Contribution	Limitations
Polge et al. [21]	2021	Hyperledger Fabric, Quorum, Ethereum, R3 Corda, and MultiChain.	Compared five major blockchain frameworks and suggested lessons for industrial practitioners and researchers for choosing frameworks.	Old versions of frameworks were used in experiments.
Thota et al. [20]	2020	Hyperledger Fabric.	Proposed software wallet to enhance security in enterprise blockchain applications and give end users consent.	Very less interoperability.
Ban et al. [19]	2019	Hyperledger Fabric.	Qualitative and quantitative comparison made between different Hyperledger flavors for the technical programmers.	Cost effectiveness and power consumption are not discussed.
Nadir [18]	2019	Permissioned blockchain leading houses; Fabric, Corda, and Quorum.	Comparison made among Fabric, Corda and Quorum blockchain and determined the best adaptable among them as Fabric.	No block size, creation time discussed.
Lasisi and Hsu [17]	2019	The consensus among public and private blockchain.	The research concluded the rationale behind the consensus among public and private blockchain.	Modular Hyperledger Fabric architecture not discussed.
Saraf and Sabadra [16]	2018	Ethereum, Corda and Hyperledger flavors; Burrow, Fabric, Indy, Iroha, and Sawtooth.	Compared different blockchains and recognized Hyperledger best for security purposes, Ethereum blockchain for decentralization and Corda blockchain for financial agreements.	Performance metrics like block size, creation time, power neglected.
Risius and Spohrer [15]	2017	Technical applicability of blockchain.	Proposed the research framework for blockchain systems and avenues for multi-disciplinary blockchain research.	Technical aspects ignored.
Vukolić [14]	2017	Architectural limitations of Hyperledger Fabric.	Proposed the redesign of HLF architecture which introduces flexible endorsement policies and pluggable modules.	No mechanism provided for chaincode generation from policies.

**Table 2:** Literature review on consensus mechanisms for IoT.

Authors	Year	Approach	Contribution	Limitations
Li et al. [31]	2021	Practical byzantine fault tolerance (PBFT).	Proposed PBFT consensus mechanism based on punishment and rewards.	RS erasure code made storage an efficient compute-intensive solution.
Wang et al. [30]	2021	Routing in unmanned aircraft systems (UAS).	Proposed lightweight proof of traffic (PoT) consensus mechanism.	Security vulnerabilities of PoT are not discussed significantly.
Raghav et al. [29]	2020	Probabilistic consensus approach.	Proposed proof of elapsed work and luck (PoEWAL).	Lacks in-depth analysis on security resistance against cyberattacks.
Biswas et al. [28]	2020	Enterprise grade business blockchain.	Proposed lightweight proof of block and trade (PoBT) consensus mechanism.	Proposed mechanism not addressed public blockchain.
Khalid et al. [27]	2020	Authentication mechanism based on fog computing.	Efficient as compared to traditional blockchain authentication techniques.	Authentication mechanisms in private blockchain are not discussed.
Huang et al. [26]	2019	Directed Acyclic Graph based blockchain.	Proposed credit-based consensus mechanism.	Security threats due to the asynchronous nature of the consensus mechanism.
Cao et al. [25]	2019	Illustrated core behind PoW, PoS and DAG consensus mechanisms.	Identified challenges and applicability of DAG-based consensus mechanisms.	Very few directions on how DAG-based consensus methods could be made suitable for IoT networks.
Dorri et al. [24]	2017	Smart homes with HPC miners.	Lightweight and efficient consensus eliminating PoW.	Vulnerable due to PoW exclusion.
Yeow et al. [23]	2017	Analysis of the applicability of blockchain consensus mechanisms in IoT.	Identified deficiencies in centralized systems and challenges in IoT enabled blockchain systems.	Very few insights on consensus mechanisms related to permissioned blockchain.
Christidis and Devetsikiotis [22]	2016	Consensus via the white-list scheme.	Proposed lightweight consensus mechanism.	Security concerns due to preselected consensus nodes.

**Table 3:** Raft algorithm modules and their corresponding improved versions.

Algorithm title	Modified version	Brief description
Initialization of the blockchain network at the start. (Leader Selection phase)	Algorithm-1.	At the start, the Raft protocol initializes the variables on the local storage and variables on volatile storage. The selection process is started on startup, leader failure, or selection timeout to elect a new leader. The leader asks the *k* random follower nodes for the vote, starts the selection timer, and upon receiving the VoteRequestMsg, those followers disseminate the message to further *k* random follower nodes.
Voting on a candidate node to become a new leader. (Leader Selection phase)	Algorithm-2.	Upon receiving VoteRequestMsg, the follower node first checks its log consistency with the candidates' log, then checks its term with the candidates' term, if both okay then follower disseminates the VoteRequestMsg message to *k* random follower nodes and VoteResponseMsg with true vote is returned, otherwise, VoteResponseMsg with false vote is returned to candidate which means candidate has outdated log.
Leader node collecting votes from the follower nodes. (Log Replication phase)	Algorithm-3.	Upon receiving VoteResponseMsg as true, the candidate node checks if its term is the same as the term of the follower node and then updates the votesReceived list, checks if it has received the quorum votes. If successful then it transits to ‘leader' state, cancels the selection timer and disseminate the message to *k* random nodes and call ReplicateLogMsg function for them.
Broadcasting the messages to the follower nodes. (Log Replication phase)	Algorithm-4.	Upon receiving the write request at the follower node it forwards that request to the leader node. Leader appends the record to its log, updates its ackedEntriesLength and calls ReplicateLogMsg function for *k* random follower nodes to disseminate the message. Leader also periodically sends heartbeats by calling ReplicateLogMsg function with empty entries for the each of the follower nodes.
Replicating from the leader node to the follower nodes. (Log Replication phase)	Unchanged.	ReplicateLogMsg function checks the sentEntriesLength of the follower node and collects the suffix of log entries ahead from the sentEntriesLength and sends the LogRequestMsg message with required information to the follower node so it could update its log with the new entries.
Followers receiving the messages from the leader node. (Log Replication phase)	Algorithm-5.	Upon receiving LogRequestMsg, the follower node checks the log consistency and term, then calls AppendEntriesMsg function to update the entries in the log of the follower. Also updates the ackedEntriesLength, send entries to *k* random nodes by calling ReplicateLogMsg function and sends LogResponseMsg to the leader with true result otherwise sends LogResponseMsg with false result to ask for old log entries from the leader.
Updating the logs of the follower nodes. (Log Replication phase)	Unchanged.	AppendEntriesMsg function checks the log of the follower for inconsistent entries and truncates them. After truncating, AppendEntriesMsg function adds the new entries to the prefix of the log of the follower and commits the entries as dictated by the leader and deliver the message to the IoT client SDK.
Leader receiving the log acknowledgments from the follower nodes. (Log Replication phase)	Unchanged.	Upon receiving LogResponseMsg as true from the follower, the leader updates the sentEntriesLength and ackedEntriesLength of that follower and calls CommitLogEntries function. On receiving LogResponseMsg as false the leader calls ReplicateLogMsg function with one previous entry so the log of the follower becomes consistent with the log of the leader.
Leader committing the log entries. (Log Replication phase)	Unchanged.	Upon receiving the acknowledgments from the quorum of nodes, the leader commits the entries in its log, after committing, the log entries become immutable. Even the leader could not remove the entries from the ledger afterward. Ultimately the leader updates its committedLength variable, and on the next heartbeat, the leader node will pass this value as an argument so follower nodes could also commit their log entries.

**Table 4:** Software and hardware system configurations.

Software & Hardware Environment	Version
Server Machine (PC)	Intel ® CoreTM i5-6500 Processor, 8gb Memory, 512gb Hard disk
Operating System	Ubuntu 18.04
Hyperledger Fabric	2.1
Docker Engine	18.06.0
Hyperledger Caliper	0.4.0

## Data Availability

The project source code and dataset are available at https://github.com/anasbaigmughal/fabric-main-mraft.

## Abbreviations

API: Application PROGRAMMING INTERFACE
CA: Certificate authority
CFT: Crash fault tolerance
DAG: Directed acyclic graph
DLT: Distributed ledger technology
FIFO: First In, First Out
GPS: Global Positioning System
HPC: High performance computing
ICT: Information and communications technology
IoT: Internet of Things
mRAFT: Modified Reliable, Replicated, Redundant, And Fault Tolerant
MSP: Membership service provider
PBFT: Practical byzantine fault tolerance
PKI: Public key infrastructure
PoBT: Proof of block and trade
PoC: Proof of capacity
PoEWAL: Proof of elapsed work and luck
PoS: Proof of stake
PoT: Proof of traffic
PoW: Proof of work
RAFT: Reliable, Replicated, Redundant, And Fault Tolerant
REST: Representational state transfer
RS: Reed-Solomon
SDK: Software development kit
SUT: System under test
UAS: Unmanned aircraft system.
